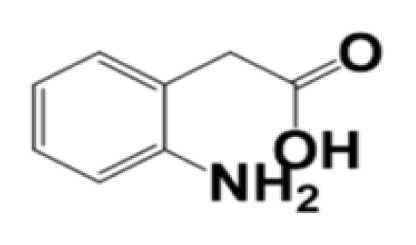# [H]-mediated enhancement of the Fenton reaction over a Pd/UiO-66(Zr)-F_4_ catalyst

**DOI:** 10.55730/1300-0527.3798

**Published:** 2026-04-25

**Authors:** Juanhong LI, Suqin WANG, Xin LIU, Feng LIU

**Affiliations:** 1 Environmental Engineering Technology Department, School of Inspection and Testing Certification, Changzhou Vocational Institute of Engineering, Changzhou, P.R. China; 2 Institute of Environmental Protection Application Technology, Collaborative Innovation Center of Water Treatment Technology & Material, School of Environmental Science and Engineering, Suzhou University of Science and Technology, Suzhou, P.R. China

**Keywords:** Regeneration of ferrous iron, hydrogen promoted Fenton reaction, UiO-66(Zr)-F_4_, nano Pd^0^ particle, carbamazepine

## Abstract

A [H]-accelerated catalytic Fenton system was developed using Pd/UiO-66(Zr)-F_4_ as the catalyst. This system enabled the regeneration of Fe^2+^ and facilitated the continuous generation of both ·OH and ^1^O_2_ through [H]-mediated electron transfer at ambient temperature and pressure. Under the conditions of 0.2 g L^−1^ catalyst (Pd loading 0.516 wt%), 25 μM Fe^2+^, 60 mL min^−1^ H_2_ flow (as the [H] source), 20 mM H_2_O_2_, and pH 3, approximately 89% of carbamazepine (initial concentration 20 mg L^−1^) was degraded within 3 h using trace iron. However, the catalytic activity of Pd/UiO-66(Zr)-F_4_ gradually declined from 89% to 53% over six reaction cycles. This decrease was likely due to loss of specific surface area caused by microstructural degradation associated with hydrogen spillover effects.

## 1. Introduction

Under normal temperature and pressure, the Fenton reaction is usually the preferred technique to degrade almost all refractory organics, except those containing C–F bonds. Currently, it is the representative advanced oxidation process in practical applications because of its easy operation and relatively inexpensive base materials, consisting of ferrous iron and H_2_O_2_ [1–5]. However, due to the much higher consumption rate of ferrous iron than its regeneration rate during the process, the Fe^II^ should be continuously used to maintain the decomposition of H_2_O_2_ and production of the reactive species represented by hydroxyl radicals (·OH) to degrade the recalcitrant organics. When the effluent is adjusted to 6~9 after the reaction, the total Fe(TFe) accumulated in the system will finally be precipitated in the form of iron sludge, which is hard to dispose of. The application and promotion of the Fenton reaction is currently hindered by this [6–10].

Compared with the strategy of either using other metal materials, such as Co and Cu, or nonmetallic carbon-based materials to replace the ferrous iron, reducing the dependence on ferrous iron may be the strategy to solve the iron sludge problem in the Fenton reaction [11–19]. Accelerating the regeneration rate of ferrous iron may be the pathway for implementing this strategy, while the reduction of Fe^III^ is the basis for implementing it. Among numerous technologies, the most direct way to accept the electron may be via either the electrification or photoelectric effect [20–24]. It could also be achieved by using electron-rich substances, such as ascorbic acid and hydroxylamine hydrochloride [1,25–31], which could be used to supply the electron for the reduction of ferrous iron. Due to the strong reducibility of activated hydrogen ([H]) with only one electron outside the nucleus as well as its reduction product being a proton, using it as the electron donor could make the technology sustainable and environmentally friendly [32,33]. After introducing the [H], derived from the H_2_ activated by Pd^0^, into the Fenton reaction system, the rapid mineralization of methyl-tert-butyl ether could be achieved without the production of iron sludge [34]. However, with a short lifetime, the [H] is not easy to store.

Improving the utilization efficiency of Pd^0^ to enhance the production of [H] may be a solution. Based on experience in chemical catalytic processes, dispersing and loading the catalytic active center is a feasible option. Considering the application scenarios under normal temperature and pressure, metal organic framework (MOF) materials with high porosity as well as surface adjustable functional groups may be ideal materials that combine catalytic carrier and hydrogen storage performance [35–39]. Rapid mineralization of emerging recalcitrant drugs such as 4-chlorophenol, sulfamethazine, carbamazepine, and trimethoprim in water could be achieved with only a trace amount of total Fe [30,40–42] in this hydrogen-promoted Fenton system enhanced by MOF materials named the MOF-hydrogen accelerated catalytic Fenton (MHACF) system.

According to the theory of soft and hard acids [42–47], UiO-66(Zr), which could be relatively stable, was screened. Particularly noteworthy is that the MHACF-UiO-66(Zr) system constructed with UiO-66(Zr) exhibited unprecedented excellent stability for the first time in the degradation of sulfamethazine. After six reaction cycles, the degradation efficiency of sulfamethazine remained above 98.5% [46]. To further improve the operational efficiency of the MHACF system, the utilization efficiency of H_2_, seen as the precursor, must be improved. Based on the surface functional group modification characteristics of UiO-66(Zr), the introduction of a fluorine group could improve the affinity of this material to gas [48,49]. However, the introduction of F may cause a slight contraction of the UiO-66(Zr) framework structure, leading to a reduction in the effective pore size. In addition, a higher degree of fluorination may increase the distortion of the aromatic rings in the ligands, which may affect the local order of the framework. Moreover, the F may occupy effective space within the pores, restricting the entry of other molecules through steric hindrance effects [50–52]. The combined effect of these factors may reduce the specific surface area of the matrix material. That is not conducive to the loading of Pd^0^ particles. How should this contradiction be reconciled? In the present work, the MHACF-UiO-66(Zr)-F_4_ system was created at the right moment to clarify this issue. The nano Pd^0^ was placed onto the outer surface of the matrix material. As a representative emerging pollutant, carbamazepine was used as the representative pollutant because it is difficult for it to be rapidly degraded by the traditional treatment process of wastewater treatment plants [53–55].

Multiple advanced technologies, including scanning electron microscopy (SEM), transmission electron microscopy (TEM), Fourier transform infrared spectroscopy (FT-IR), X-ray diffraction (XRD), and analysis of surface area and pore size distribution, were used to characterize the solid catalytic materials. The removal of pollutant was elucidated by control experiments. The evolution process of active particles was determined by detection of degradation products, conducting quenching experiments coupled with the analysis of electron spin resonance (ESR). The durability of the composite catalysts was also examined through six reaction cycles. Our work provides a novel perspective for peers to study advanced oxidation processes and expand the application of both MOF materials and hydrogen in the treatment of environmental pollution in future.

## 2. Materials and methods

### 2.1. Chemicals

Both palladium(II) chloride and hydrogen peroxide (H_2_O_2_), of analytical grade, were supplied by China National Pharmaceutical Group Corporation. Methanol and acetonitrile, of HPLC grade, were obtained from Thermo Fisher Scientific Co., Ltd. All other chemicals were sourced from Shanghai Aladdin Biochemical Technology Co., Ltd. and were used as received without further purification. Ultrapure water (Millipore Milli-Q system, resistivity >18.2 MΩ cm) was used for the preparation of all solutions.

### 2.2. Synthesis of catalyst

UiO-66(Zr)-F_4_, which was the matrix material in the present work, was synthesized to serve as the catalyst support for the active center. The synthesis procedure was as follows: a mixture of 48 mL of ultrapure water and 32 mL of acetic acid was prepared in a beaker. Subsequently, 1.84 g of ZrCl_4_ and 1.19 g of tetrafluoroterephthalic acid were added to the solution, which was then subjected to ultrasonication for 30 min. The resulting mixture was transferred to a Teflon-lined stainless steel autoclave and heated at 383 K for 24 h in a drying oven. After cooling, the product was washed three times alternately with anhydrous methanol and anhydrous DMF. The solid was then dried under vacuum at 423 K. Prior to use, the material was activated under vacuum at 423 K for 2 h.

The preparation of Pd/UiO-66(Zr)-F_4_, which was the catalytic material used in our work, was carried out as described previously [40,41]. In brief, the synthesis strategy was as follows. Firstly, the activated hydrogen generated from the decomposition of sodium borohydride in water was used to reduce palladium dichloride to nano Pd^0^ particles. Subsequently, the as-prepared UiO-66(Zr)-F_4_ was added under ice-water bath conditions with stirring, thereby yielding Pd/UiO-66(Zr)-F_4_.

### 2.3. Experimental procedures

The error bars displayed in the figures indicate the standard deviation derived from triplicate experimental measurements. All tests were performed under ambient temperature and pressure conditions. The mixing of solutions was done by magnetic stirrer. Two-necked flat bottom flasks were used to conduct the reaction at room temperature. To prevent light exposure, the outer surfaces of the flasks were wrapped with aluminum foil. The H_2_ used as the source of [H] was produced by an SPH-300 hydrogen generator (Beijing BCHP Analytical Technology Institute, P.R. China). Before the start of the experiment, H_2_ was continuously supplied into solution over 5 min in order to eliminate dissolved oxygen. Ferrous iron, solid material, and H_2_O_2_ were added sequentially. With the adding of H_2_O_2_, the reaction started. At specified reaction time points, liquid samples were extracted and filtered using 0.22-μm PTFE syringe filters (Tianjin Keyilong Experimental Equipment Co., Ltd, P.R. China). One part of them was quenched at once by using one drop of wood alcohol, and then for the assay of carbamazepine, ferrous iron and H_2_O_2_. The remaining filtrate, which was not quenched with CH_3_OH, was utilized for the analysis of total Pd (TPd), total Zr (TZr), and total organic carbon (TOC). Upon completion of each robustness test cycle, the spent catalyst was recovered by filtration through a glass frit medium. The recovered solid was washed three times with ultrapure water and dried overnight under vacuum at 423 K before being reused in the subsequent reaction cycle.

### 2.4. Methods of analysis

The test center of Yangzhou University provided most of the characterization services for solid catalysts. The models of the corresponding instruments are not repeated herein because they have been mentioned many times previously [40,43,56]. The specific surface area was determined through N_2_ adsorption–desorption measurements performed at 77 K. Before analysis, all samples were degassed under vacuum at 378 K for 24 h. Analysis of the contact angle of the material was conducted with a contact angle meter made in China (JC2000D6, Shanghai Zhongchen Digital Technology Equipment Co., Ltd). The reactive species were identified by the ESR (A300-10/12, Bruker Corporation, Germany) test. 5,5-Dimethyl-1-pyrroline N-oxide (DMPO) was employed as the trapping agent for ·OH, O_2_·^−^ and activated hydrogen, while 2,2,6,6-tetramethyl-4-piperidinone (TEMP) was used to detect ^1^O_2_.

The concentration of carbamazepine was analyzed using a Shimadzu LC-20AT high-performance liquid chromatography system equipped with an Agilent ZORBAX Eclipse XDB-C_18_ column (4.6 mm × 150 mm, 5 μm) [54]. Degradation intermediates were identified by high-performance liquid chromatography-mass spectrometry (HPLC-MS; LTQ, Thermo Fisher Scientific, USA) [57,58]. TOC content in aqueous solutions was determined with a Multi N/C 3100 analyzer (Analytik Jena, Germany). Inorganic anions were quantified using a Dionex ICS-900 system (Thermo, USA) with a conductivity detector, an IonPac AS23 analytical column, and an IonPac AG23 guard column [40,43,56]. Ammonia, H_2_O_2_, and ferrous ions were measured via spectrophotometric methods [40,42]. Total metal ion concentrations were analyzed by inductively coupled plasma optical emission spectrometry (ICP-OES) on an Agilent 720ES instrument (USA).

## 3. Results and discussion

### 3.1. Characterization of experimental materials

The detailed characterization results of UiO-66(Zr), as the matrix material of the MHACF-UiO-66(Zr) system that previously demonstrated excellent performance in the degradation of sulfamethazine, are found in our previous work [46].

**Figure 1:**
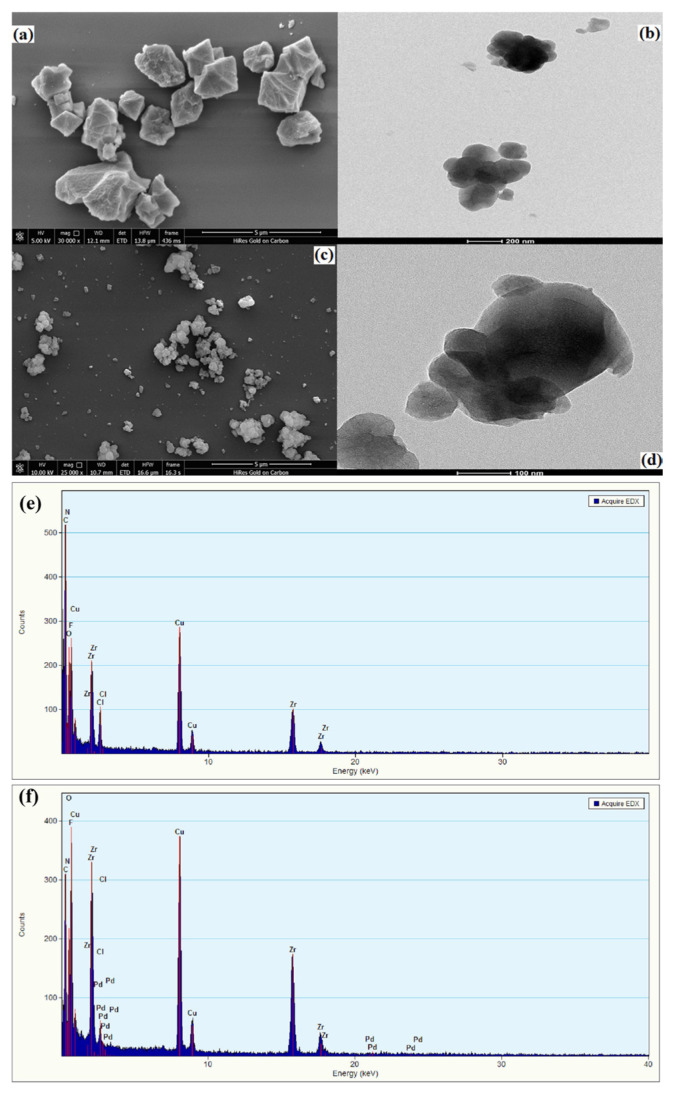
(a) and (b) SEM and TEM photographs of synthesized matrix material; (c) and (d) SEM and TEM photographs of synthesized composite material; (e) EDX of synthesized matrix material; (f) EDX of synthesized composite material.

According to Figures 1a and 1b, the morphology of the matrix material presented an inconspicuous pyramid-shaped polygonal structure. The average diameter of particles ranged from 180 nm to 200 nm. That was the same as the diameter of UiO-66(Zr) [59,60]. According to Figures 1c and 1d, no significant morphology or structure change was observed after loading the Pd^0^ particles onto the outer face of the matrix material. According to Figures 1e and 1f, the Pd element was detected in the synthesized composite material. Nano Pd^0^ particles were observed, as seen in Figures 1c and 1d. The Pd content of composite material was approximately 0.52% as measured using ICP-OES. The measured water contact angle value of UiO-66(Zr) was 22.91°. This value for the synthesized matrix material was 41.23° and 38.10°, respectively. This indicated that the hydrophobicity of the fluorinated material had increased.

**Figure 2:**
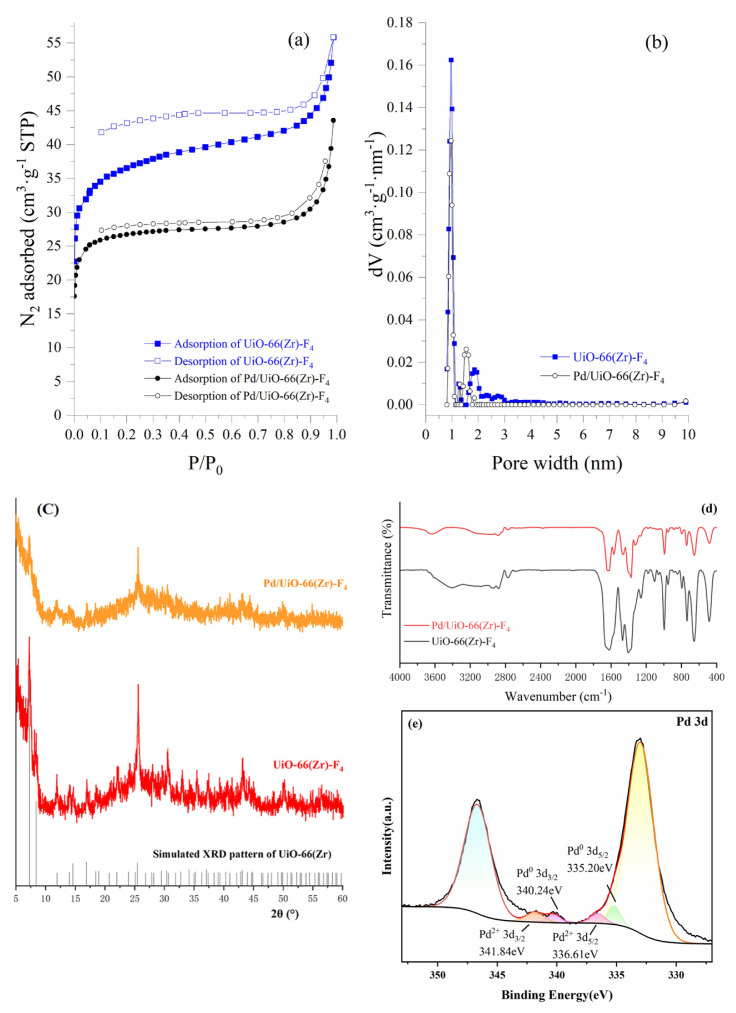
(a) N_2_ adsorption–desorption isotherms of materials; (b) the pore size distribution curves of materials; (c) XRD pattern of materials; (d) FT-IR spectra of materials at wavenumbers ranging from 4000 to 500 cm^−1^; (e) the XPS Pd 3d spectra of synthesized composite material.

According to Figure 2a, the synthesized matrix material exhibited a type IV adsorption–desorption isotherm, as did the synthesized composite material. Compared with UiO-66(Zr) with a specific surface area of over 1000 m^2^ g^−1^ [46], the surface area of the synthesized matrix was only 136.90 m^2^ g^−1^. As known, the introduction of fluorine may cause a slight contraction of the UiO-66(Zr) framework, resulting in a reduced effective pore size. In addition, a higher degree of fluorination may increase the distortion of the aromatic rings within the ligands, which could adversely affect the local order of the framework. Furthermore, fluorine atoms may occupy effective space inside the pores, thereby restricting the entry of other molecules through steric hindrance effects [50–52]. The combined effect of these factors may result in a decrease in the specific surface area of the substrate material following fluorination. The surface area of the composite material was 104.76 m^2^ g^−1^. The slight decrease in this value after loading the palladium once again could be used to prove that the nano Pd^0^ particles had invaded the surface of the matrix material. The unclosed adsorption–desorption curve implied a relatively narrow and complex pore size distribution structure. Combined with the pore size distribution exhibited in Figure 2b, although it still maintained the main microporous structure, other mesoporous structures were still widely present. This result was similar to previous related research reports [59–61]. The composite material showed comparable results. From the XRD pattern presented in Figure 2c, it can be seen that the synthesized matrix material retained the basic crystal structure of UiO-66(Zr). This was consistent with the results reported for UiO-66(Zr)-F_4_ [52,59,60,62]. The main diffraction peaks in the XRD pattern of the composite material were generally consistent with those of the matrix material, while their intensities were significantly reduced. At the same time, many diffraction peaks, such as the peaks at 8.42°, 20.73°, 21.99°, 35.25°, 39.09°, 48.06°, and 56.10°, had disappeared. This indicated that the degree of amorphousness of the composite material was increased. As illustrated in Figure 2d, the absorption peak at 996 cm^−1^ corresponds to the C–F bending vibration [62]. The absorption peak at 1625 cm^−1^ corresponds to the C=O stretching vibration. The signal at 1104 cm^−1^ is indicative of C–O stretching, while those at 736 cm^−1^ and 655 cm^−1^ are characteristic of Zr–O vibrations. These results confirmed the incorporation of Zr–O clusters and C–F into the UiO-66(Zr)-F_4_ framework [40,63–67]. However, in contrast, the intensities of all peaks in the FT-IR spectrum of the composite material, especially the peak corresponding to C–F, had weakened or disappeared to varying degrees. Combined with the XRD results mentioned above, this indicated that the operation of nano Pd^0^ particle loading may affect the structural stability of the matrix material to some extent. As seen in Figure 2e, the results showed that relevant diffraction peaks of Pd could be clearly observed in the synthesized composite material, which was consistent with the results of EDX. The diffraction peak signal of Pd species could hardly be observed, probably due to the low loading of Pd, which was similar to the results of the ICP test and XRD spectrum. The weaker signal-related diffraction peaks belonging to Pd 3d_5/2_ and Pd 3d_3/2_ were observed at 335.20 eV and 340.24 eV. Combined with the results of EDX, the ICP test, and XRD spectrum, it could be proved that the nano Pd^0^ particles had been successfully loaded into the structure of synthesized matrix material [68].

Collectively, these results confirm the successful synthesis of both UiO-66(Zr)-F_4_ and Pd/UiO-66(Zr)-F_4_.

### 3.2. Valence state cycling of Fe in the MHACF-UiO-66(Zr)-F4 system

**Figure 3:**
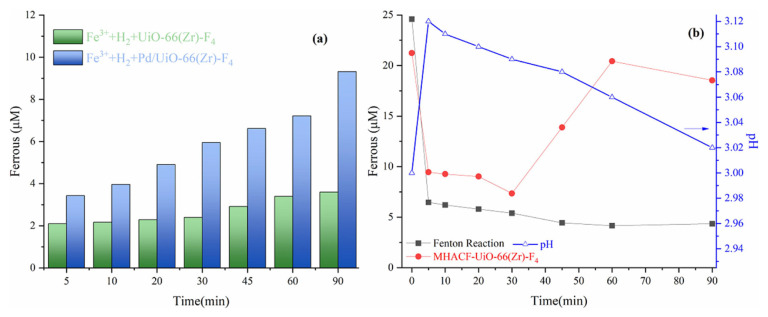
The Fe^II^/Fe^III^ redox cycle in the reaction system: (a) Variation profiles of ferrous iron in the Fe^III^-H_2_-Pd/UiO-66(Zr)-F_4_ system, Fe^III^-H_2_-UiO-66(Zr)-F_4_ system, respectively (initial pH 2.5); (b) Variation profiles of ferrous iron and pH in the MHACF-UiO-66(Zr)-F_4_ system, and variation profile of ferrous iron in the Fenton system. Except for the parameters investigated, other initial parameters were FeCl_3_ 25 μM, Pd/UiO-66(Zr)-F_4_ 0.2 g L^−1^ (Pd content 0.52% (W_Pd_:W _Pd/UiO-66(Zr)-F4_), H_2_ 60 mL min^−1^, ferrous iron 25 μM, H_2_O_2_ 20 mM, and pH 3.

The Fe^III^ will not be reduced even if H_2_ is directly introduced unless it is converted into [H] [40–44]. Both the MOF materials with hydrogen storage properties and nano Pd^0^ particles could be used to activate H_2_ into [H] [45–47]. As shown in Figure 3a, after 90 min of reaction in the UiO-66(Zr)-F_4_+H_2_ system, about 3.6 μM of ferrous iron was detected, while 9.3 μM of ferrous iron was detected in the Pd/UiO-66(Zr)-F_4_+H_2_ system. These results suggest that Fe^II^ was regenerated through reduction by [H]. As the electronic carrier, the Fe^II^ was regenerated by the continuously reduction electron supplied by [H], while as the by-product, the proton was accumulated within the system [34]. It may cause the pH of the MHACF system to show an overall downward trend. The inference was confirmed by the variation in pH in Figure 3b. As the reduction proceeded, the consumption of ferrous iron in the MHACF-UiO-66(Zr)-F_4_ system was significantly slower than that in the Fenton reaction. Moreover, the pH of the MHACF system showed an overall downward trend. This result indicated that Pd/UiO-66(Zr)-F_4_ effectively promotes the reduction of Fe^III^.

### 3.3. Performance of the MHACF-UiO-66(Zr)-F4 system

**Figure 4:**
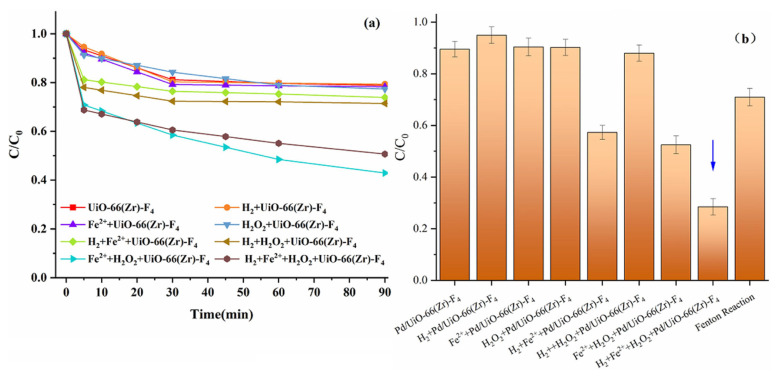
The control test. (a) The removal of carbamazepine by UiO-66(Zr)-F_4_ (without loading Pd^0^) under various conditions; (b) The removal of carbamazepine by Pd/UiO-66(Zr)-F_4_ under various conditions compared with the Fenton reaction after 90 min of reaction. Except for the parameters investigated, other initial parameters was ferrous iron 25 μM, UiO-66(Zr)-F_4_ 0.2 g L^−1^, Pd/UiO-66(Zr)-F_4_ 0.2 g L^−1^ (Pd content 0.52% (W_Pd_:W_Pd/UiO-66(Zr)-F4_)), H_2_ 60 mL min^−1^, H_2_O_2_ 20 mM, carbamazepine 20 mg L^−1^, and pH 3.

Based on previous findings [30,67], the contributions of ferrous coagulation, H_2_O_2_ oxidation, and H_2_ stripping to carbamazepine removal were found to be negligible under ambient temperature and pressure conditions. As shown in Figure 4a, in the absence of nano Pd^0^ particles, the removal efficiency of carbamazepine—attributable only to trace reactive oxygen species produced via the Fenton reaction—was below 22%. This value remains higher than the adsorption efficiency of the matrix material alone, which was less than 11%. The low removal efficiency could be attributed to the lack of [H] generated by Pd^0^ activation in the reaction [34].

After introducing nano Pd^0^ particles, this situation could be significantly improved within the same reaction time. As seen in Figure 4b, the Pd/UiO-66(Zr)-F_4_ contributed less than 11% to the adsorption of carbamazepine. Its removal efficiency was a maximum of 11%. The reactive oxygen species that could be used to degrade carbamazepine was not enough through the active in situ decomposition of H_2_O_2_ [46,68,69]. It could be significantly improved after further introducing a trace amount of ferrous iron. It could be about 41% and 34% in the Fe^2+^ + H_2_O_2_ + Pd/UiO-66(Zr)-F_4_ and Fe^2+^ + H_2_ + Pd/UiO-66(Zr)-F_4_ system, respectively, after 90 min of reaction, while it was still less than 12% in the H_2_ + H_2_O_2_ + Pd/UiO-66(Zr)-F_4_ system. It could be about 72% in the MHACF-UiO-66(Zr)-F_4_ system while it was only about 30% in the Fenton reaction. The TOC concentration declined from 19.75 mg L^−1^ to 15.87 mg L^−1^, indicating effective degradation of carbamazepine in the MHACF-UiO-66(Zr)-F_4_ system.

The organic degradation products detected after 90 min of reaction are shown in the Table. It indicated that the degradation of carbamazepine was related to its hydroxylation and deamidation, both of which were related to the ·OH. At the same time, the degradation process also yielded detectable amounts of ammonia (2.60 mg L^−1^), nitrate (9.60 mg L^−1^), and nitrite (0.13 mg L^−1^). Together with the preceding results, these data further verify the degradation of carbamazepine within the MHACF-UiO-66(Zr)-F_4_ system.

### 3.4. Operational mechanism of the MHACF-UiO-66(Zr)-F4 system

**Figure 5:**
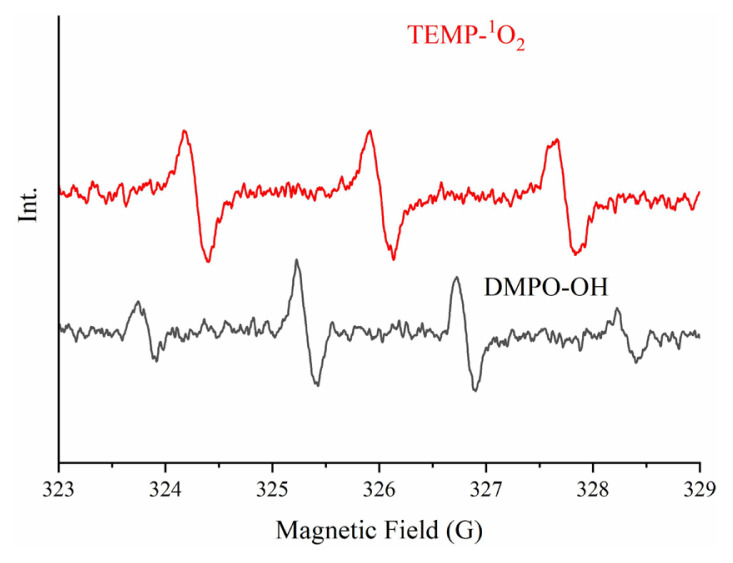
EPR spectra for the observation of ·OH and ^1^O_2_ in the MHACF-UiO-66(Zr)-F_4_ system. The initial parameters were scavenger 100 mM, pH 3, Pd/UiO-66(Zr)-F_4_ 0.2 g L^−1^ (Pd content 0.52% (W_Pd_:W_Pd/UiO-66(Zr)-F4_)), H_2_O_2_ 20 mM, H_2_ 60 mL min^−1^, ferrous 25 μM, reaction time 5 min.

**Figure 6:**
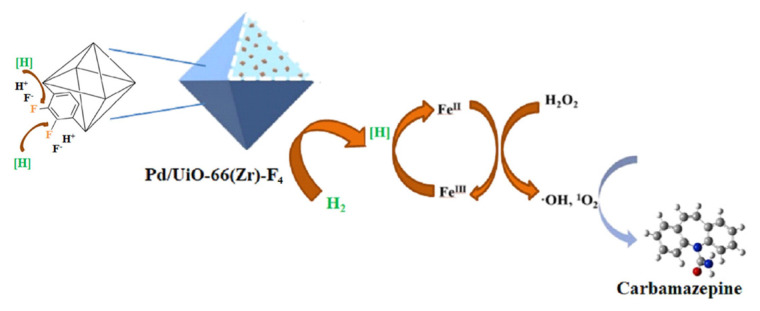
Operation of the MHACF-UiO-66(Zr)-F_4_ system.

According to Figure 3, the ferrous iron was regenerated in this system. Although no significant signal of active hydrogen was observed in the ESR experiment, this may be attributed to the rapid consumption of active hydrogen [68,69]. According to Figure 5, ·OH and ^1^O_2_ were detected in the ESR test while the typical signals of O_2_^•−^ [70,71] could not be detected. Although the absence of O_2_^•−^ only indicated that its content was below the detection limit, the presence of [H] could also be used to ensure the normal evolution between the two main reactive oxygen species. The [H] could be regarded as a proton carrying only one outer electron. Due to the outer layer having only one electron, it exhibits reducibility. This enabled the continuous reduction of Fe^III^ in the MHACF-UiO-66(Zr)-F_4_ reaction, thereby maintaining the continuous production of ·OH (Eqs. 1–3) [34]. In addition, either Pd^0^ (Eqs. 4 and 5) [34,46] or [H] (Eq. 6) [72] can also achieve the effect of activating H_2_O_2_. All of them ensured the continuous generation of ·OH. Adequate ·OH can not only degrade pollutants through “wolf pack tactics”, but also convert them into ^1^O_2_ (Eq. 7) [73]. Under the combined action of ·OH and ^1^O_2_, the carbamazepine could be rapidly degraded, as seen in Figure 6.


(Eq. 1) $${\text{Fe}}^{\text{II}}+{\text{H}}_{2}{\text{O}}_{2}\to {\text{Fe}}^{\text{III}}+\cdot \text{OH}+{\text{OH}}^{-}$$

(Eq. 2) $${\text{H}}_{2}+2{\text{Pd}}^{0}\to 2\text{Pd}-[\text{H}]$$

(Eq. 3) $${\text{Fe}}^{\text{III}}+\text{Pd}-[\text{H}]\to {\text{Pd}}^{0}+{\text{Fe}}^{\text{II}}{+}^{\text{H}+}$$

(Eq. 4) $${\text{O}}_{2}+2\;\text{Pd}-[\text{H}]\to 2{\text{Pd}}^{0}+{\text{H}}_{2}{\text{O}}_{2}$$

(Eq. 5) $${\text{H}}_{2}{\text{O}}_{2}\to 2\cdot \text{OH}$$

(Eq. 6) $$\text{Pd}-[\text{H}]+{\text{H}}_{2}{\text{O}}_{2}\to \cdot \text{OH}+{\text{H}}_{2}\text{O}+{\text{Pd}}^{0}$$

(Eq. 7) 4·OH→O12+2H2O

### 3.5. Robustness of the MHACF-UiO-66(Zr)-F_4_ system in carbamazepine degradation

**Figure 7:**
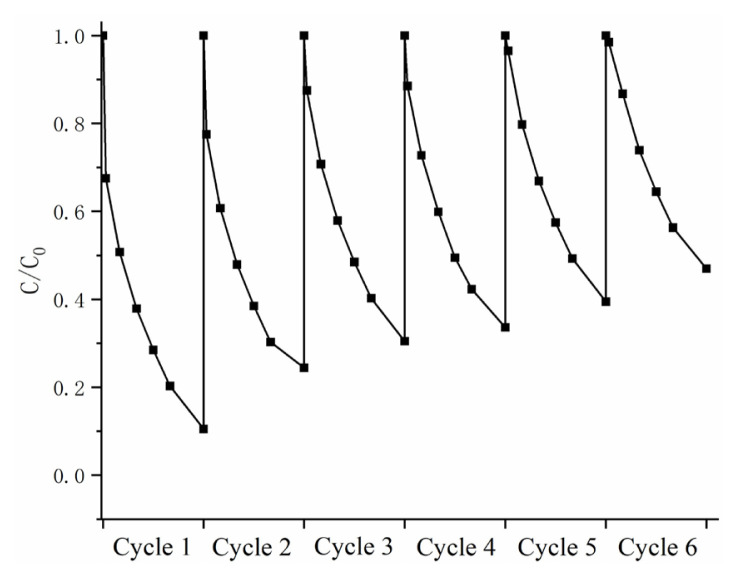
Removal of carbamazepine in the MHACF-UiO-66(Zr)-F_4_ system during the six consecutive reaction cycles. The parameters were Fe^2+^ 25 μM, H_2_O_2_ 20 mM, Pd/UiO-66(Zr)-F_4_ 0.2 g L^−1^ (Pd content 0.52% (W_Pd_:W_Pd/UiO-66(Zr)-F4_)), H_2_ 70 mL min^−1^, initial carbamazepine 20 mg L^−1^, and initial pH 3.

Each cycle of the robustness test for the MHACF-UiO-66(Zr)-F_4_ system was conducted over 180 min to achieve more comprehensive degradation of carbamazepine. After six reaction cycles, as shown in Figure 7, its removal efficiency was 89.51% (1st cycle), 75.57% (2nd cycle), 67.32% (3rd cycle), 66.38% (4th cycle), 60.52% (5th cycle), and 53.03% (6th cycle). Although this result may seem acceptable, compared to the stable and long-lasting effect of MHACF-UiO-66(Zr) on the degradation of sulfamethazine [46], the stable and efficient application effect on the MHACF system was not improved by the improvement in hydrophobicity caused by surface fluorination.

**Figure 8:**
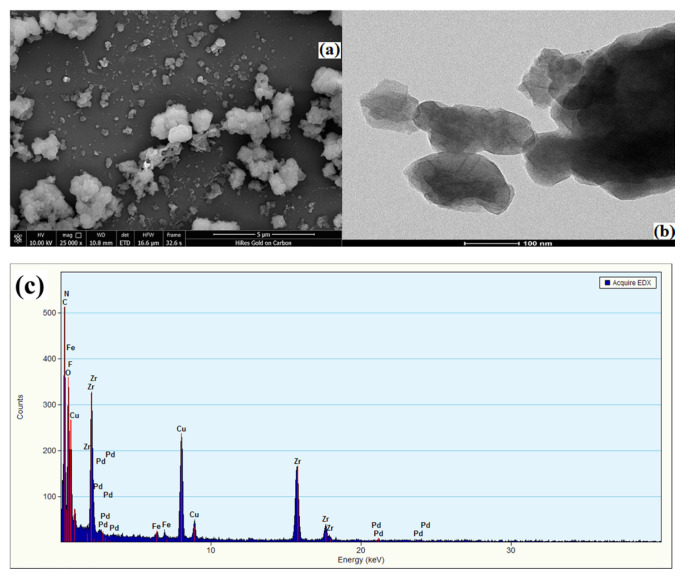
SEM, TEM, and EDX photographs of the residual Pd/UiO-66(Zr)-F_4_ after six reaction cycles.

As depicted in Figure 8, the morphology of Pd/UiO-66(Zr)-F_4_ after six reaction cycles shows notable alterations compared to the freshly prepared material presented in Figure 1. The originally distinct sharp-edged particles were no longer detectable and the field was predominantly occupied by fragmented, irregularly shaped aggregates.

**Figure 9:**
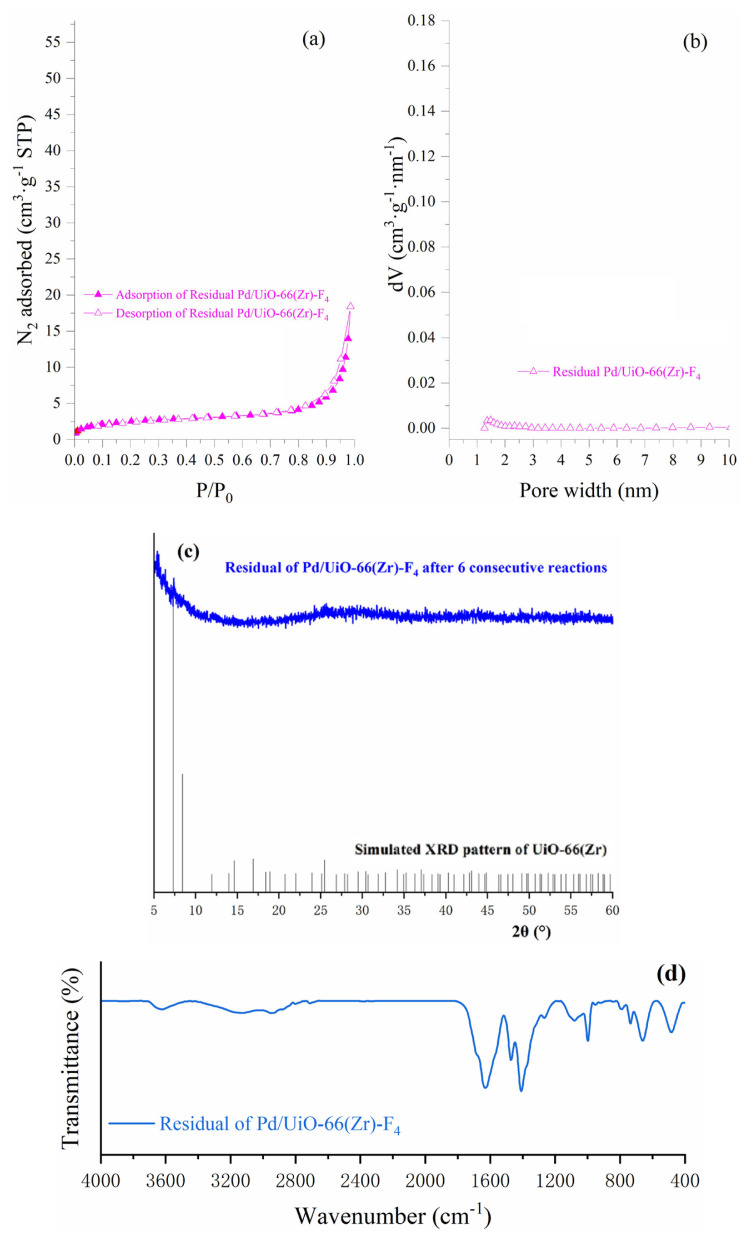
(a) N_2_ adsorption–desorption isotherms of the residual Pd/UiO-66(Zr)-F_4_; (b) the pore size distribution curves of residual Pd/UiO-66(Zr)-F_4_; (c) the XRD pattern of residual Pd/UiO-66(Zr)-F_4_; (d) FT-IR spectra of residual Pd/UiO-66(Zr)-F_4_ at wavenumbers ranging from 4000 to 500 cm^−1^.

As shown in Figures 9a and 9b, although the isotherm type of the spent Pd/UiO-66(Zr)-F_4_ remained largely unchanged, its specific surface area decreased to 9.14 m^2^ g^−1^, accompanied by near-complete disappearance of the microporous structure. Although neither TZr nor TPd was detected throughout the reaction cycles, for the composite material after six reaction cycles, compared with the XRD pattern of the freshly prepared composite, the diffraction peaks, as displayed in Figure 9c, had almost completely disappeared. Moreover, its peaks of FT-IR—particularly that corresponding to C–F—were weakened, as seen in Figure 9d. The composite material after six reaction cycles had become nearly amorphous. These changes suggested a notable alteration in the fundamental structure of UiO-66(Zr)-F_4_. Furthermore, the concentration of F^−^ in the MHACF-UiO-66(Zr)-F_4_ system increased from 0 to 3.24 mg L^−1^ after 1 h of reaction. In contrast, no F^−^ release was detected in control experiments using freshly prepared Pd/UiO-66(Zr)-F_4_ added to aqueous solution (initial pH 3), 20 mM H_2_O_2_ (pH 3), 25 μM ferrous solution (pH 3), or the Fenton system (pH 3, 20 mM H_2_O_2_, 25 μM Fe^2+^). It could be confirmed that the F was reduced and detached from the benzene ring. As known, even those with strong oxidation ability like ·OH cannot destroy the C–F with the bond energy of 485 kJ mol^−1^, while it can be defluorinated through an electron-donating reduction reaction [74]. The [H] could be regarded as a proton with an outer electron. This structure results in its strong reducibility. Therefore, it could be used in the reduction of C–F. The hydrogen storage performance of MOF materials could be promoted by the hydrogen spillover effect [75,76]. Therefore, the F could be reduced and detached during the migration of [H] on the surface of UiO-66(Zr)-F_4_. This would lead to the instability of matrix material. It could also be used to further explain why, after loading the nano Pd^0^ particles, the degree of amorphousness of the matrix material increased, as seen in Figure 2c. Further, the C–F structure was damaged. This was because the preparation of nano Pd^0^ particles involved the use of sodium borohydride, a strong reducing agent that reduces Pd^2+^ through the activated hydrogen generated in water [40,41]. After the reduction process, the residual sodium borohydride remaining could also react with water to produce activated hydrogen. That may damage part of the structure of the matrix material by reducing C–F, as illustrated in Figures 2c and 2d. In the present work, it was maintaining the specific surface area of catalyst, which seems to be more important than regulating its hydrophobicity in the MHACF system.

## 4. Conclusion

The MHACF system was constructed using UiO-66(Zr)-F_4_ in the present work. Nano Pd^0^ particles were deposited on the external surface of UiO-66(Zr)-F_4_. The carbamazepine, chosen as the target pollutant, was quickly degraded by ·OH and ^1^O_2_ with trace amounts of TFe. Although the hydrophobicity of matrix material was improved to a certain extent after fluorination, [H] caused the reduction and detachment to the C–F, resulting in a decrease in the specific surface area. Based on this analysis result, the improvement of the MHACF system in future research could be conducted by the regulation of hydrophobicity through modifying the electron-donating group onto the UiO-66(Zr). We hope to provide colleagues who are committed to improving advanced oxidation processes with some different perspectives through it.

## Figures and Tables

**Table:** Organics detected in the solution after 90 min of reaction in the MHACF-UiO-66(Zr)-F_4_ system.

m/z	Molecular Structures
271	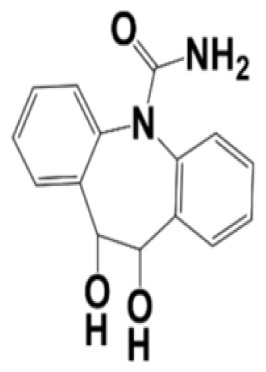
253	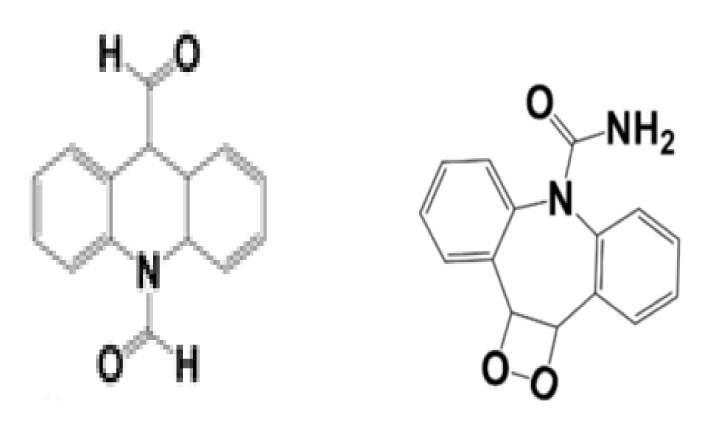
239	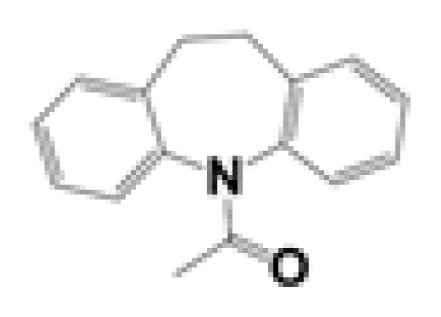
236	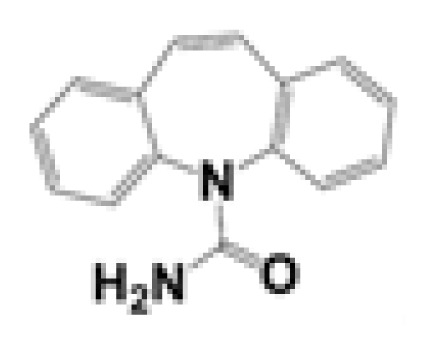
152